# The Continual Reassessment Method for Multiple Toxicity Grades: A Bayesian Model Selection Approach

**DOI:** 10.1371/journal.pone.0098147

**Published:** 2014-05-29

**Authors:** Haitao Pan, Cailin Zhu, Feng Zhang, Ying Yuan, Shemin Zhang, Wenhong Zhang, Chanjuan Li, Ling Wang, Jielai Xia

**Affiliations:** 1 School of Statistics, Xi'an University of Finance and Economics, Xi'an, China; 2 Department of Gastrointestinal Surgery, Xijing Hospital of Digestive Diseases, Fourth Military Medical University, Xi'an, China; 3 Department of Hematology, Shandong Provincial Hospital Affiliated to Shandong University, Jinan, China; 4 Department of Biostatistics, The University of Texas MD Anderson Cancer Center, Houston, Texas, United States of America; 5 Department of Health Statistics, Fourth Military Medical University, Xi'an, China; National Institute of Environmental and Health Sciences, United States of America

## Abstract

Grade information has been considered in Yuan et al. (2007) wherein they proposed a Quasi-CRM method to incorporate the grade toxicity information in phase I trials. A potential problem with the Quasi-CRM model is that the choice of skeleton may dramatically vary the performance of the CRM model, which results in similar consequences for the Quasi-CRM model. In this paper, we propose a new model by utilizing bayesian model selection approach – Robust Quasi-CRM model – to tackle the above-mentioned pitfall with the Quasi-CRM model. The Robust Quasi-CRM model literally inherits the BMA-CRM model proposed by Yin and Yuan (2009) to consider a parallel of skeletons for Quasi-CRM. The superior performance of Robust Quasi-CRM model was demonstrated by extensive simulation studies. We conclude that the proposed method can be freely used in real practice.

## Introduction

The primary goal of a phase I clinical trial of a new oncologic agent is to find a dose with acceptable toxicity, that is, to target the maximum tolerated dose (MTD). In practice, the MTD is often defined as the dose of the drug that will produce a defined dose-limiting toxicity(DLT) in a pre-specified percentage of patients. Toxicity level is often categorized into multiple grades. For instance, the general guidelines of the Common Toxicity Criteria (CTC) (National Cancer Institute,2003) are grade 0 for no toxicity; grade 1,2,3,4 and 5 for minimal toxicity, moderate toxicity, severe toxicity, life threatening and death, respectively. This comprehensive toxicity grading scale is well-established and adopted in clinical practices, which indicates that a binary response may inappropriately ignore kinds of levels of toxicity severity. However, in most dominating dose allocation approaches, such as the traditional "3+3" design [1], CRM design [2] and recently proposed mTPI design [3], these grades are dichotomized. For example, if grade 4 fatigue is considered DLT then grades 0–3 will be non-DLT and treated almost equally from the point of view of a clinical trial design. It is known that such dichotomization works well for moderate toxicities. Nevertheless, for severe and possibly irreversible effects, such as renal, liver, or neurological toxicities, grade 4 renal toxicity is much more severe than that of grade 3. Therefore, those toxicity grades should not be treated indiscriminately. In addition, given that Phase I trials are typically small in size, utilizing as much information as possible for decision making is important. Using only partial toxicity information could be inefficient. More appropriate methods need to be used to consider this issue in the dose escalation procedure.

In the literature, there have been some proposals for considering this issue. Bekele and Thall (2004) [4] (BT method for short) applied severity weights to a soft tissue sarcoma trial with five types of DLTs. Each observed patients was assigned by physicians to a severity weight on a common numeric scale for each type of toxicity, and the sum of these weights over the five toxicity types was called the total toxicity burden (TTB). The authors then considered a hypothetical collection of cohorts with a variety of different possible outcomes. Yuan, Chappell and Bailey (2007) [5] (their proposed method is named as Quasi-CRM) also used severity weights to convert toxicity grades to numerical scores. They proposed a Quasi-CRM approach to incorporate these scores into the CRM. The recommended dose for the next patient is the dose level with estimated score (the equivalent toxicity (ET) score) closest to the target score, obtained from a pre-specified toxicity profile at the MTD. This Quasi-CRM method has been demonstrated to be superior to the BT method in recommendation percentage of optimal dose for further studies. Meter, Garrett-Mayer and Bandyopadhyay (2012) [6] incorporated toxicity grades using a continuation ratio (CR) model in the likelihood-based CRM. They demonstrated that the proposed method was better than that of dichotomous CRM counterpart.

In 2009, Yin and Yuan [7] proposed using multiple parallel CRM models, each with a different set of pre-specified toxicity probabilities. In the Bayesian paradigm, they assign a discrete probability mass to each CRM model as the prior model probability. The posterior probabilities of toxicity can be estimated by the Bayesian model averaging (BMA) approach. Dose escalation or de-escalation is then determined by comparing the target toxicity rate and the BMA estimates of the dose toxicity probabilities. Yin and Yuan examine the properties of the BMA-CRM approach through extensive simulation studies, and also compare this new method and its variants with the original CRM. The results demonstrate that the BMA-CRM is competitive and robust, and eliminates the arbitrariness of the pre-specification of toxicity probabilities. However, the BMA-CRM approach does not take the multiple toxicity grade level into account.

Although the Quasi-CRM method has good statistical performance, as in the CRM paradigm, the Quasi-CRM method only uses a pre-specified skeleton for the estimation of parameters, which could induce unstable estimators according to Yin and Yuan (2009). In this article we inherit the essence of BMA-CRM approach to incorporate it into the Quasi-CRM paradigm. We call our proposed design Robust Quasi-CRM. Numeric comparisons of Robust Quasi-CRM, Quasi-CRM are described in Section 3, followed by a conclusion in Section 4.

## The Method

### 1. Equivalent Toxicity Score

The "equivalent toxicity (ET) score" was proposed by Yuan et al.(2007) to measure the relative severity of different toxicity grades in the dose allocation procedure, where grade 3 toxicities are assigned a value of 1, grades 2 are assigned to 0.5, and grades 4 are assigned to 1.5. They consider an ET score equal to 1 as the cutoff grade for DLT. By introducing the concept of ET score, the commonly used MTD definition will be modified to incorporate grade information. A new MTD is defined as the dose of the drug with ET score equal to the target ET score, computed from a pre-specified toxicity grade profile at the MTD. Please also refer to Bekele and Thall (2004) for details.

### 2. Quasi-CRM

The quasi-likelihood function is constructed using a family of probability distributions that may not contain the true distribution. Estimators obtained by maximizing the quasi-likelihood function are called quasi maximum likelihood estimates (QMLEs). Under some regularity conditions, QMLEs are strongly consistent if the "quasi" distributions belong to linear exponential families such as the binomial family (Gourierox, Monfort, and Trognon, 1984; McCullagh and Nelder, 1989) [8] [9].

Suppose that n patients have been tested sequentially at dose levels d(1), d(2), 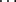, d(n) with corresponding ET scores s(1), s(2), 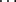, s(n). Define the normalized ET scores as
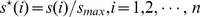
where 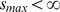. Thus 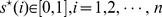. The normalized scores can be viewed as fractional events and modeled using the quasi-Bernoulli likelihood (Papke and Woodbrige, 1996) [10]. Obviously, their true distributions are not Bernoulli, which only takes values of 0 or 1. However, if the dose-toxicity model is correctly specifed, the QMLE will be strongly consistent because the Bernoulli distribution belongs to the binomial family.

Assume that the true dose-normalized ET score relationship is given by 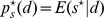. Consider J dose levels 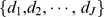. Denote 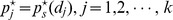. The goal is to find the MTD 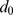 that is the highest level such that 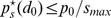, where 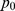 is the target ET score. Assume that the last patient is tested at level 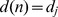 with normalized ET score 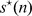. Then its contribution to the quasi-Bernoulli likelihood will be
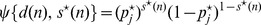



The quasi-Bernoulli likelihood will be updated by 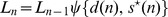, and 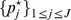 can be estimated accordingly. Note that if a functional dose-score curve is not assumed, the QMILE 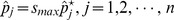, equals to the observed average ET score at each dose level.

The quasi-Bernoulli likelihood provides a simple way to incorporate ordinal grades into parametric models. Yuan et al.(2007) successfully used it with the CRM in developing the Quasi-CRM.

### 3. Bayesian Model Selection Method

As pointed out by Yin and Yuan (2009), a major issue associated with the CRM is that pre-specification of the toxicity probabilities 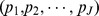 is arbitrary. If the 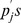 deviate far from the true dose-toxicity curve, this may lead to poor operating characteristics and a high probability of selecting the wrong dose as the MTD. To avoid subjectivity in specifying the skeleton, they proposed pre-specifying multiple skeletons, each representing a set of prior estimates of the toxicity probabilities. During the trial, conditional on the observed data, these different models usually yield different estimates of the toxicity probabilities 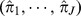. Some of these estimates may be close to the true values, whereas others may not, depending on how well the models fit the accumulated data. To accommodate the uncertainty in the specification of these skeletons, Yin and Yuan (2009) took a BMA approach to average 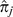 across the CRM models to obtain the BMA estimate of the toxicity probability for dose level j. BMA is known to provide a better predictive performance than any single model (Raftery, Madigan, and Hoeting 1997; Hoeting et al.1999) [11] [12].

Specifically, let 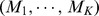 be the models corresponding to each set of prior guesses of the toxicity probabilities 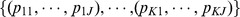. Model 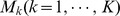 in the CRM is given by
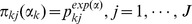
which is based on the kth skeleton 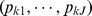. Let 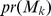 be the prior probability that model 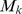 is the true model; that is, the probability that the kth skeleton 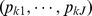 matches the true dose-toxicity curve. If there is no preference a priori for any single model in the CRM case, then one can assign equal weights to the different skeletons by simply setting 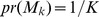. At a certain stage of the trial, based on the observed data 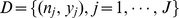, the likelihood function under model 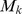 is



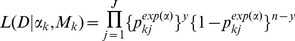



The posterior model probability for 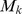 is given by
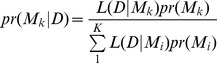
where 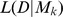 is the marginal likelihood of model 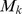, 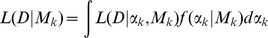, 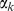 is the power parameter in the CRM associated with model 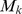, and 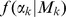 is the prior distribution of 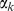 under model 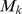.

The posterior model probability can be naturally linked to the Bayes factor, which also consequently resulting in the Bayesian model selection approach. The Bayes factor, 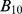, for model 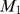 against another model 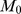 given data D is defined as the ratio of posterior to prior odds, 
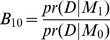
, which is the ratio of the marginal likelihood, i.e., 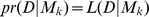. We can construct such Bayes factors for each of the models 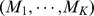 against 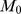, denoted by 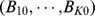. Then the posterior model probability of 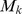 is
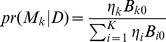
where 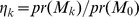 is the prior odds for 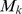 against 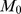, 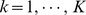.

The Bayesian model selection approach can be used to estimate the toxicity probabilities and make the decision of dose assignment. Specifically, at each point of decision making for dose assignment, we select the model with the highest posterior probability, i.e., model
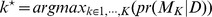
and use that model to make inference and dose assignment.

Unlike the Quasi-CRM, our proposed robust version pre-specifies a parallel of 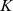 different skeletons, 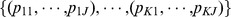. Then after n patients, the quasi-posterior estimation of the toxicity probability for dose j under the 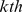 skeleton will be updated by
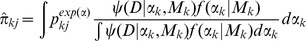
here we use the quasi-Bernoulli likelihood 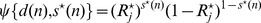.

According to the BMA-CRM approach, in our proposed method, we also add a stopping rule in our algorithm, that is, if 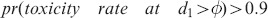, then the trial is terminated for safety. We give our proposed approach the name *Robust Quasi-likelihood approach* in later section. Here, we require early termination of a trial if the lowest dose is too toxic, as noted by
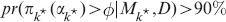



## Simulation studies

### 1. Simulation settings

We investigated the operating characteristics of the proposed *Robust Quasi-likelihood approach* through simulation studies under eight different toxicity scenarios. Table 1, the same as in Yuan et al., gave the probability configurations for grades 0–4 in each scenario. We considered six dose levels and assumed that toxicity increased monotonically with respect to the dose. We prepared three sets of initial guesses of the toxicity probabilities:
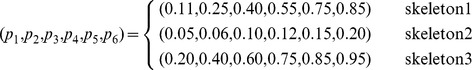



**Table 1:** True probabilities of each grade (0/1,2,3,4) at each dose level (1–6) for eight simulation scenarios (A–H).

Scenario	Grade	Dose 1	Dose 2	Dose 3	Dose 4	Dose 5	Dose 6
A	0,1	0.83	0.75	0.62	0.51	0.34	0.19
	2	0.12	0.15	0.18	0.19	0.16	0.11
	3	0.04	0.07	0.11	0.14	0.15	0.11
	4	0.01	0.03	0.09	0.16	0.35	0.59
B	0,1	0.92	0.85	0.70	0.55	0.24	0.00
	2	0.03	0.05	0.10	0.15	0.26	0.36
	3	0.03	0.07	0.14	0.21	0.35	0.49
	4	0.02	0.03	0.06	0.09	0.15	0.21
C	0,1	0.78	0.56	0.50	0.40	0.30	0.16
	2	0.14	0.19	0.18	0.17	0.15	0.09
	3	0.06	0.12	0.14	0.15	0.14	0.10
	4	0.02	0.12	0.18	0.28	0.41	0.65
D	0,1	0.88	0.64	0.52	0.35	0.17	0.00
	2	0.04	0.12	0.16	0.22	0.28	0.39
	3	0.06	0.17	0.22	0.30	0.38	0.52
	4	0.02	0.07	0.10	0.13	0.17	0.23
E	0,1	1.00	0.91	0.88	0.86	0.80	0.65
	2	0.00	0.06	0.07	0.08	0.10	0.13
	3	0.00	0.03	0.04	0.05	0.08	0.14
	4	0.00	0.00	0.01	0.01	0.02	0.08
F	0,1	0.50	0.38	0.29	0.19	0.13	0.08
	2	0.25	0.24	0.21	0.16	0.11	0.07
	3	0.11	0.12	0.12	0.10	0.08	0.05
	4	0.14	0.26	0.38	0.55	0.68	0.80
G	0,1	0.78	0.58	0.50	0.40	0.30	0.16
	2	0.14	0.18	0.18	0.17	0.15	0.09
	3	0.00	0.00	0.00	0.00	0.00	0.00
	4	0.08	0.24	0.32	0.43	0.55	0.75
H	0,1	0.92	0.76	0.68	0.57	0.45	0.25
	2	0.00	0.00	0.00	0.00	0.00	0.00
	3	0.08	0.24	0.32	0.43	0.55	0.75
	4	0.00	0.00	0.00	0.00	0.00	0.00

The first skeleton started at a relatively moderate toxicity probability of 0.11 and increased quickly at the high toxicity probability of 0.85. The second skeleton was for the case in which toxicity increased slowly at the low doses but increased quickly at the moderate doses; the highest dose had a toxicity probability of 0.20. The toxicity probabilities in the third skeleton were scattered evenly over a range of 0.2 to 0.95. Thus these three sets of skeletons represented three different prior opinions on the true dose-toxicity curve. We refered to the individual Quasi-CRMs using each of these three skeletons as Quasi-CRM 1, Quasi-CRM 2, and Quasi-CRM 3.

In Table 2, under each scenario we listed the true toxicity probabilities in the first row, the corresponding ET scores in the second row, the percentages of MTD dose being correctly identified and the average numbers of patients treated at each dose separately for the Quasi-CRM using each of the three skeletons in rows 3–8, the results obtained using the proposed Robust Quasi-CRM in rows 9–10.

**Table 2:** Recommendation percentage for each dose under Scenarios A–H.

	Recommendation percentage at dose level						
Design	1	2	3	4	5	6	#of Toxicity
Scenario A							
(ET score)	0.12	0.19	0.34	**0.48**	0.76	1.05	
Quasi-CRM 1	0	0.043	0.415	**0.493**	0.048	0.001	
# patients	1.702	3.370	7.165	**6.627**	1.074	0.062	1.136
Quasi-CRM 2	0.08	0.068	0.349	**0.364**	0.199	0.012	
# patients	1.455	2.175	4.964	**8.062**	3.146	0.198	3.344
Quasi-CRM 3	0.002	0.116	0.488	**0.380**	0.014	0.000	
# patients	1.941	4.787	7.860	**4.810**	0.598	0.004	0.602
Robust Quasi-CRM	0.001	0.066	0.378	**0.466**	0.060	0.029	
# patients	1.936	3.718	5.682	**6.567**	1.945	0.152	2.097
Scenario B							
(ET score)	0.08	0.14	0.28	**0.42**	0.70	0.98	
Quasi-CRM 1	0.0	0.019	0.245	**0.620**	0.116	0.0	
# patients	1.483	2.479	5.387	**8.357**	2.269	0.025	2.294
Quasi-CRM 2	0.002	0.030	0.240	**0.403**	0.314	0.011	
# patients	1.334	1.697	3.593	**8.353**	4.766	0.257	5.023
Quasi-CRM 3	0.0	0.041	0.327	**0.547**	0.085	0.0	
# patients	1.584	3.077	6.443	**7.409**	1.487	0.0	1.487
Robust Quasi-CRM	0.0	0.022	0.214	**0.569**	0.158	0.037	
# patients	1.584	2.480	4.609	**7.647**	3.423	0.257	3.68
Scenario C							
(ET score)	0.0	0.06	0.09	0.10	0.16	**0.32**	
Quasi-CRM 1	0.0	0.0	0.001	0.050	0.235	**0.714**	
# patients	1.034	1.228	1.474	2.882	5.571	**7.811**	
Quasi-CRM 2	0.0	0.0	0.0	0.0	0.015	**0.985**	
# patients	1.002	1.064	1.114	1.526	4.995	**10.299**	
Quasi-CRM 3	0.0	0.0	0.011	0.090	0.547	**0.352**	
# patients	1.029	1.360	2.191	4.330	8.101	**2.989**	
Robust Quasi-CRM	0.0	0.0	0.001	0.0	0.088	**0.902**	
# patients	1.025	1.209	1.399	1.620	4.288	**10.519**	
Scenario D							
(ET score)	**0.44**	0.63	0.80	1.01	1.16	1.29	
Quasi-CRM 1	**0.700**	0.267	0.032	0.001	0.0	0.0	
# patients	**13.480**	5.031	1.296	0.171	0.021	0.001	6.52
Quasi-CRM 2	**0.738**	0.212	0.048	0.002	0.0	0.0	
# patients	**12.545**	4.985	1.943	0.467	0.060	0.0	7.455
Quasi-CRM 3	**0.744**	0.232	0.024	0.0	0.0	0.0	
# patients	**14.366**	4.534	0.956	0.137	0.007	0.0	5.634
Robust Quasi-CRM	**0.731**	0.249	0.016	0.001	0.002	0.001	
# patients	**13.984**	4.548	1.175	0.258	0.032	0.003	6.016
Scenario E							
(ET score)	0.16	0.40	**0.50**	0.66	0.83	1.12	
Quasi-CRM 1	0.031	0.477	**0.379**	0.106	0.007	0.0	
# patients	3.380	8.465	**5.717**	2.055	0.368	0.003	2.426
Quasi-CRM 2	0.088	0.337	**0.379**	0.153	0.041	0.002	
# patients	3.108	5.647	**5.957**	4.065	1.140	0.083	5.288
Quasi-CRM 3	0.045	0.578	**0.308**	0.066	0.003	0.0	
# patients	4.011	9.671	**4.872**	1.340	0.106	0.0	1.446
Robust Quasi-CRM	0.038	0.473	**0.359**	0.109	0.014	0.007	
# patients	4.099	7.791	**4.854**	2.515	0.676	0.065	3.256
Scenario F							
(ET score)	0.11	0.34	**0.45**	0.60	0.78	1.06	
Quasi-CRM 1	0.006	0.281	**0.480**	0.226	0.007	0.0	
# patients	2.383	6.291	**7.208**	3.746	0.369	0.003	4.118
Quasi-CRM 2	0.038	0.206	**0.457**	0.224	0.072	0.003	
# patients	2.182	4.403	**6.397**	5.539	1.395	0.084	7.018
Quasi-CRM 3	0.005	0.388	**0.470**	0.131	0.006	0.0	
# patients	2.533	8.026	**6.818**	2.395	0.227	0.001	2.623
Robust Quasi-CRM	0.009	0.321	**0.447**	0.189	0.024	0.010	
# patients	2.607	6.880	**6.273**	3.418	0.785	0.037	4.240
Scenario G							
(ET score)	0.19	**0.45**	0.57	0.73	0.90	1.17	
Quasi-CRM 1	0.125	**0.508**	0.302	0.064	0.001	0.0	
# patients	5.070	**8.206**	4.799	1.683	0.234	0.008	6.724
Quasi-CRM 2	0.188	**0.403**	0.296	0.084	0.028	0.001	
# patients	4.630	**6.167**	5.189	2.995	0.965	0.054	9.203
Quasi-CRM 3	0.130	**0.561**	0.269	0.037	0.003	0.0	
# patients	5.140	**8.927**	4.562	1.249	0.122	0.0	5.933
Robust Quasi-CRM	0.104	**0.531**	0.271	0.075	0.011	0.008	
# patients	5.160	**7.861**	4.302	2.045	0.585	0.047	6.979
Scenario H							
(ET score)	0.08	0.24	0.32	**0.43**	0.55	0.75	
Quasi-CRM 1	0.0	0.065	0.327	**0.451**	0.149	0.008	
# patients	1.797	3.866	6.041	**5.722**	2.279	0.295	2.574
Quasi-CRM 2	0.001	0.035	0.182	**0.300**	0.383	0.099	
# patients	1.449	2.215	3.987	**6.816**	4.694	0.839	5.533
Quasi-CRM 3	0.001	0.120	0.388	**0.376**	0.113	0.002	
# patients	1.780	4.624	6.624	**5.304**	1.637	0.031	1.668
Robust Quasi-CRM	0.001	0.097	0.243	**0.360**	0.202	0.097	
# patients	1.858	4.018	4.697	**5.614**	2.961	0.852	3.813

In the simulations, the target ET score was 0.47, which is equivalent to DLT probability of 0.33. That is, if we consider the following toxicity profile: 49% grade 0 and grade 1, 18% grade 2, 23% grade 3, and 10% grade 4, then the target ET score was obtained by computing the weighted sum of ET scores over all grades (i.e., 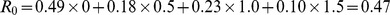.) All simulations began at the lowest dose and cohorts of one were treated at each stage. Dose escalation was restricted to the next higher pre-specified dose only. Each scenario was simulated 1,000 times with a maximum sample size of 20.

### 2. Simulation results

In scenario A, the fourth dose was the desirable dose, and the three individual Quais-CRMs using different skeletons selected the targeted ET score with very different probabilities. In particular, the proposed Robust Quasi-CRM correctly identified the MTD 46.6% of the time. Quasi-CRM 1 performed the best, correctly identifying the MTD 49.3% of the time and the Quasi-CRM 2 performed the worst, only correctly identifying the MTD 36.4% of the time. In this case, while the Quasi-CRM design was slightly better than the Robust Quasi-CRM, they were very comparable both in terms of correctly identifying the MTD as well as with respect to the number of subjects who were treated above the MTD.

Scenario B had the MTD at the fourth dose level, and the MTD selection percentage using the Robust Quasi-CRM was the second best among the four designs. The worst skeleton corresponds to Quasi-CRM 2, only correctly identifying the MTD 40.3% of the time, whereas the proposed design correctly identified the MTD 56.9% of the time. In scenario C, the sixth dose was the MTD. Quasi-CRM 3 performed the worse in this scenario, with a MTD being correctly identified almost 50% lower than those of the others. In this case, Quasi-CRM 1's performance was also inferior to that of the proposed Robust-CRM method.

In Scenario D, the first dose is the MTD. Skeleton 1 correctly identified the MTD 70% of the time, while the proposed Robust-CRM correctly identified the MTD 73.1% of the time. With respect to the number of patients assigned to the above target ET score, our Robust design is the second best. Scenario E is similar to scenario A. In scenario F, all of the percentages of the MTD being correctly identified by using different designs were quite close, except the Quasi-CRM 2 has assigned more patients to the above target ET score than others. In scenarios G and H, again the proposed Robust Quasi-CRM was very robust, with a MTD selection percentage always close to that of the best-conducted Quasi-CRM.

These findings demonstrate that the skeleton indeed plays a critical role in the Quasi-CRM design. There was a difference of 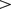 55% in the MTD selection probability when using different skeletons in scenario E. However, our Robust Quasi-CRM performed the second best, with MTD being identified around 90.2% of the time.

Based on these simulations, we conclude that the proposed Robust Quasi-CRM method are quite robust in terms of dose selection probabilities and average number of patients treated at the MTD level. **These methods typically cannot perform as well as the best single Quasi-CRM in the skeletons set, but their performance is always quite close to that of the best single Quasi-CRM and can be much better than that of the worst single Quasi-CRM.** The proposed method carries the essence of the BMA-CRM proposed by Yin and Yuan (2009) by adaptively balancing among competing models, and thus offers more reliable and robust estimates for the toxicity probabilities.

## Conclusion

In this paper we proposed the robust version of Quasi-CRM to model toxicity grades, and demonstrated by simulation that it is superior to the single skeleton version of Quasi-CRM. As pointed by Yuan et al.(2007), the Quasi-CRM is most useful when DLTs are severe, possibly irreversible, or have a long duration.

The performance of the proposed designs can be substantially improved over that of the original Quasi-CRM if the skeleton in the CRM happens to be very far from the true model. The Robust Quasi-CRM method is straightforward to implement and to compute easily based on the Gaussian quadrature approximation or the Markov Chain Monte Carlo procedure. Our method requires specifying multiple skeletons to cover different potential scenarios for the underlying dose-toxicity curve. It provides a nice compromise for the initial guesses of toxicity probabilities from different physicians. If one skeleton corresponds to the true toxicity probabilities, then the Robust Quasi-CRM would perform very well, because it often performs similarly to the best-performing Quasi-CRM. This Bayesian model-averaging procedure dramatically improves the robustness of the Quasi-CRM. As shown in the simulations, a certain skeleton often yields under-performing results; however, simultaneously specifying multiple skeletons reduces the likelihood of all sets of toxicity probabilities leading to a poorly performing Quasi-CRM design. The arbitrariness in the specification of the skeleton is eliminated by incorporating the uncertainties associated with each skeleton into the Bayesian model-averaging procedure.

In our simulations we used a cohort size of one; however, cohort size of two or three also could be used. Our setup is based on the improved versions of the Quasi-CRM to optimize its practical performance. As an extension of the Quasi-CRM, the Robust Quasi-CRM makes this trial design more widely applicable and reliable for phase I clinical trials.
